# On the Origin and Progress of Empiricism

**Published:** 1805-11-01

**Authors:** Duncan Forbes


### ON THE ORIGIN AND PROGRESS OF EMPIRICISM.

BY DUNCAN FORBES, M.D.

THE sentiments and character of man appear to be much influenced by those social institutions with which he is conversant. In dark and barbarous times, he sinks into abject slavery, and becomes unable to extricate himself from the fetters of prejudice; but in the progress of civilization, he gradually acquires a greater confidence in his powers, and assumes a higher tone in his pretensions. He attaches himself to the cultivation of science; knowledge begins to be more widely diffused, and the fine arts receive encouragement and protection. It is nevertheless a galling consideration, that while other branches of human knowledge are progressing towards maturity, the science of medicine, which confers upon mankind the greatest of all comforts, except those of religion, should be among the last of those that attain to perfection. The healing art, consigned among Savages to the charms of the reputed sorcerer, is by a large proportion of the inhabitants of civilized states, but too frequently intrusted to the interested pretensions of nefarious quacks, and to the far less dangerous prescriptions of superannuated females; while gross ignorance, servile complaisance, criminal duplicity, and meanness of spirit, constitute prominent features in the characters of not a few of our regular practitioners both in town and country.

In the opinion of the populace, the medical practitioner ought to possess a specific remedy, calculated to alleviate or remove every infirmity which preys upon human life. This unjust requisition on the part of the multitude confounds the empiric with the physician. The latter carefully considers the whole phenomena of the disease, and marks with attention the effects of remedies; his knowledge is of course acquired by observations, and by attending to the results of well instituted experiments. The intellectual character of the former may be thus briefly delineated. A medical empiric is one, who ignorant of the operations of nature, and disregarding her laws; ignorant too of the causes and characteristic symptoms of a disease, and a stranger to the opinions which at different periods have been entertained regarding it, and to the light reflected on it, by the observations and experience of past ages, simply asks its name, and then, without any view to rational indications of cure, administers his nostrums in random succession. Now we may fairly argue, that this man's experience must be false, and that he must inevitably plunge himself deeper and deeper in error, because his presumption leads him to practice an art, of the principles of which he is grossly ignorant; and because he is a servile imitator of other men, while he comprehends not those views of the diseases, which suggested their practice.

In the infancy of medical science, a careful inspection of diseases was necessary before they could be discriminated, or their causes explained. The empiric too is often desirous to see his patient; but then he neither investigates the case before him, nor is anxious to ascertain the *data*, on on which his practice rests. He rejects all information, proclaims open hostility against every principle, and deems himself instructed, as by heavenly inspiration, in all the *arcana* of the Esculapian school. I would not be understood as denying, that even empirics are capable of forming certain combinations; but I do not hesitate to contend, that these combinations are confined to the superficial ideas of things, or rather that they extend not beyond the perceptions of the senses.

I have somewhere read, that the physicians of Chili blow round the bed of their patients to drive away diseases. The Chilians seem firmly persuaded, that dexterity in the management of this wind, substantiates the character of an experienced practitioner; and their doctors would, no doubt, ill brook any innovation which might multiply the difficulties in the method of cure.

It must be confessed that regular physicians themselves, by exhibiting occasional marks of duplicity and meanness of spirit, by a servile compliance with the caprices of their patients, and by sacrificing to every idol of fashion, have in all ages considerably abetted the cause of empiricism. This charge can be easily substantiated by an appeal to medical history both in ancient and modern times. Egypt was the ancient repository of medical science; and until the æra of the Mamalukes, many of the Egyptian physicians were equally respectable from their literary attainments, and for the zeal and probity with which they exercised their profession. But these barbarous and ignorant tyrants requited the care and skill of their medical attendants by the most licentious and arbitrary cruelty. Sunk in the profoundest ignorance, and of course strangers to every principle which should regulate the conduct of a physician, they insisted on immediate relief from every the slightest sensation of pain. The physicians, terrified into compliance by the threatenings of despotism, and compelled to act in obedience to the blind caprice of these absolute masters, no longer aimed at rational indications of cure, but at soothing the impatience and irrascibility of their princes. Instead of investigating the principal seat of the disease, they confined their attention to some particular symptoms, which seemed to require immediate mitigation; and having thus succeeded in procuring a momentary alleviation of pain, the sources of the complaint were left inherent in the constitution, and the tyrant was abandoned to his fate. By this mode of practice, they were indeed successful in conciliating the affections of

 their their employers; but medical science rapidly degenerated, and since that period the physicians of Egypt rank among the most illiterate and superstitious empirics.

Galen, whose extensive genius and indefatigable industry enabled him to concentrate within himself, all the knowledge of nature which previous inquiry had brought to light; Galen, the tenor and spirit of whose work bespeak a mind under the best influence, who pursued the science which had been the object of his early choice and long contemplation, with extraordinary ardour and intensity of thought, through all its immediate and remote connections; and whose attention was engaged by every object possessing the most distant real or imaginary reference to medical pursuits, feelingly complains of many physicians of his day, who unblushingly made their court to the ladies, by assiduous attendance at the morning toilet, and by devoting the evening to the most sumptuous amusements. By thus countenancing the reigning follies, and modelling themselves to every fashion, they obtruded themselves on the public attention, and endeavoured to establish their reputation. And, hence the reason, adds this eminent man, why the fine arts and philosophy are considered as very useless branches of the physician's knowledge.

I know not whether the profession at this day can claim an exemption from this serious charge, brought by the excellent physician of Pergamos against the medical fops of his age; and if the charge actually attach to us, can we be surprised that ignorant mechanics should quit the lath or the loom, for the more lucrative trade of an empiric? or that the mere compounder of medicines, armed with his Pharmacopœia, his pestle, and his mortar, should boldly strut forth in the character of a physician, and undertake the treatment of diseases?

Pliny has very well observed, that he who is possessed of a sufficient stock of impudence, may easily enough pass for a physician.

The unblushing effrontery of empirics, and their success in ingratiating themselves with the many, had not escaped the attention of the great Lord Bacon, who remarks, that an impostor frequently rules supreme at a patient's bedside, and that his crude reveries are received as the decisions of an oracle; while genuine merit is insulted, and his opinions are treated with contempt, the people having always had a propensity to encourage the pretensions of quacks and old women, and to regard them as as the rivals of true physicians. The length of diseases, continues this profound student of human nature, the sweets of life, the illusive flattery of hope, and the recommendations of the patient's friends, constitute the grounds of that preference, not unfrequently given to the vilest and most ignorant quacks, over the most experienced and greatest adepts in the science. Hope, indeed, is the last friend of the distressed; and the ignorant fellow, who has studied the humour more than the disease of his patient, will encourage its indulgence, while the physician of learning and of conscience, however much his humanity may dispose him to revive his patient's drooping spirits, by every encouragement and hope which can honestly be suggested to him, will never represent the danger as less than he actually believes it to be; will never intentionally be so far misled by mistaken tenderness, as to be guilty of positive falsehood; regarding the holy apostle's injunction, not to do evil that good may come, as clear, and positive, and universal, he will never flatter the patient or his relations, by giving promises or raising expectations, which he knows and intends to be delusive.

In his competition with the empiric, the regular practitioner labours under many disadvantages. If by a kind effort of nature, any of his promises be realized by the event, the former is applauded to the skies, and a thousand voices proclaim his success; but should the patient find his disease aggravated, or his confidence misplaced, a sense of shame imposes silence, lest blame and ridicule attach to him, for having intrusted his constitution to the management of an impostor, who considers the credulity of mankind, as a licence for the exercise of his bare-faced roguery. It may further be observed, that the empiric runs no risque of shipwrecking his reputation, because the ignorant alone prop his fame, and because in every instance of failure, the blame attaches to those who listened to his absurd pretensions. The empiric further addresses himself to that predilection for novelty, and to that *penchant* for the marvellous, with which the bulk of mankind are so deeply tinctured; these being predominant, are the more amply gratified, the more the quacks pretensions exceed the bounds of rational probability. He applies a barbarous name to a plant, which he had picked up just as he entered the village, and expatiates in a pompous strain on its miraculous virtues; this plant is immediately enrolled in the list of specifics, and adopted for the cure of every infirmity.

Galen

Galen has left us a finished portrait of an empiric, in his character of Thessalus, a noted quack, in the reign of Nero. As the medical impostors of every age may, in not a few circumstances, claim kindred with Thessalus, I shall subjoin a translation, for the benefit of those of the profession, whose classical attainments are not sufficient to enable them to consult the original.

The father of Thessalus, says Galen, was a workman, who tried in vain to give his son some idea of what is great and beautiful. Without the least tincture of letters or philosophy, Thessalus took it into his head to commence physician; and according to his own gross way of thinking, he was really entitled to this appellation. He had not however proceeded far in his new career, before he felt himself deficient in many parts of knowledge, and in the qualities which are necessary to enable a man to advance with credit in his profession. He still preserved the tone, the manners, and the language of a man of trade; but through his thin disguise, you might easily distinguish in him the carder of wool. Conscious of his defects in all the requisites of his profession, he began to win upon his patients, not by prescribing remedies suitably adapted to the circumstances of their case, but by flattering their hopes, and by sacrificing to their vanity Notwithstanding the natural severity of his temper, he knew how to mould himself occasionally to the will of his patients, when he saw that his low complaisance would turn to good account. But with all his suppleness to those whose favour he had gained, he shewed the greatest impudence and temerity towards all regular practitioners; and he had no sooner succeeded at Rome by his meanness, than he exclaimed without remorse against all physicians, and even went so far as to assert, that himself was the only person who deserved that title. He was not less injurious to the dead than to the living, and even took pleasure in reviling the memory of Hippocrates.*

Such was the character of Thessalus, as delineated by a contemporary observer; and does not many an illiterate, self-consequential practitioner of our own times, strongly resemble the original picture? Is it not equally a subject of surprise and regret, that our constituted authorities should not only tolerate these destructive vermin, but even sanction

* Vide Galen Pergameni de Med. Method. Lib. prim. sub. init. vel. Epitomen. Gal. Oper. &c. Auct A. Lacuna. Fol. p. 409.

sanction their deleterious pretensions? I shall, in a future part of this essay, take the liberty of going into some detail relative to this glaring abuse of the royal prerogative, in giving currency to the sale of Panaceas, and Specifics, and Nostrums: here I shall content myself with observing, by the way, that the people, blind, and obstinate, and ignorant as they are, should not be surrendered as a prey to a set of impudent and dangerous men, who unfeelingly tamper with their lives, and fleece them of their substance. The Sovereign has been stiled, yes, and justly stiled, the father of his people, he is unquestionably a man of humanity; we are of course authorised in the supposition, that he would lend a favourable ear to any representation which might be made to him on this subject, why then should not so flagrant an evil be expostulated against, and why should not our colleges unite their joint influence in the reformation of such destructive abuses?

From the preceding sketch, it is I think obvious, that in every age, and in every country, medical impostors have contested the ground with scientific and regular physicians. We must not however deceive ourselves by the use of words, or suppose that the term *Empiricism*, which is now the designation of those who banish all reasoning and theory from the art of medicine, and maintain, that experience is the sole guide to safe and successful practice, originally paid no regard to logical deductions from theory, or rested their practice entirely on their own limited experience. That the fact was otherwise will manifestly appear from the following historical detail, relative to the rise and progress of this celebrated medical sect.

It must be obvious to remark, that in the earliest ages of physic, before philosophy was incorporated with medicine, and influenced its practice, the most rational and conscientious physicians must have been guided by their own observations, and of course must have ranked among the empirics. It is true, they had not then assumed this term, as an appropriated designation, neither had they arranged themselves into a particular sect, but they coincided in opinion as to the plan of practice to be pursued, and they scrupulously traced the footsteps of their predecessors. No sooner, however, had they enlarged the sphere of their knowledge, than they deviated from their beaten tract, and each insensibly explored for himself a different route. The greater number devoted their time to useless researches, and were wholly occupied with frivolous subtleties, resulting from the defective philosophy of the times.

These

These diversified modes of speculation necessarily resulted in different conclusions; and these conclusions as naturally suggested the employment of different remedies. Particular articles must have had more decided effects than others; and such effects coinciding with prevalent opinion, gradually gave rise to a sect, who displeased with the unavoidable mistakes of their theoretical masters, withdrew from them their whole confidence; and from an exalted sense of the preference due to observation over authority, resolved wholly to abandon the speculative subtleties of their brethren, and to rest their practice entirely on experience. This sect dates its origin from the æra of Herophilus, an eminent professor of the Alexandrian School, whose discoveries contradicted every established opinion relative to animal sensation, and who justly concluded the art of reasoning, as of inferior importance to the means of cure. Herophilus observed, that from philosophising on the causes of diseases, physicians were too frequently led to reject the most important remedies, because they did not chime with their systematic ideas; and hence the foundation of that scepticism, which led him to conclude, that physicians, in proportion to their imagined scientific acquirements, deviated from the plain path of observation and of experiment.

Philenus of Cos, the pupil of Herophilus, carried his aversion from medical speculation a step further than his master. He not only contended that such as endeavoured to penetrate into the recesses of physic lost themselves in the labyrinth of errors, but that even the anatomical knowledge which he derived from the prelections and demonstrations of his teacher, did not add to his resources in the treatment of diseases; that of consequence the attempt to explore the causes of diseases, must necessarily be unavailing, since anatomy itself furnished no light to conduct the enquirer in the path of discovery. He contended that the physician should abandon the search after hidden and undiscoverable causes, and attend to the obvious phenomena of nature; and that theory and hypothesis should give place to experience and observation. Serapion, another eminent teacher of Alexandria, reduced these ideas into a system, and became, according to Celsus, the founder of a sect, the followers of which assumed the name of empirics from the Greek word Εμπειρα, which signifies experience.

But, in order to contrast the pretensions of ancient and modern empirics, it will be necessary to attend to the import of the term *experience*, as adopted by the disciples of Philenus and Serapion. These ancient physicians, then, understood by *experience*, all the knowledge brought to light either by chance or by experiment, all the knowledge that could be deduced from a careful attention to the effects in which the same experiment, repeated in various circumstances, resulted. A Physician, in their opinion, possessed true experience, when, by means of various trials, and of various results, he was enabled to establish certain *data*, from which the effects of similar trials, in similar circumstances, might be calculated; or from which the *rationale* of such deviations from the usual results, as, on particular emergencies might occur, could be accounted for with sufficient probability. The first step towards the acquisition of this knowledge, was, in their judgment, habitual and attentive observation; the second, a careful perusal of what had been written by others, concerning the history and cure of such diseases as had been the subject of their practice. By thus combining reading with actual observation, they hoped that the physician would be able to ascertain the point of resemblance and of difference between various diseases, and thus, when any new case came under his inspection, to form a judgment as to the proper indications of cure, from recollecting the mode of practice adopted in some known disease, to which the case under deliberation bore a resemblance. This view of the subject Serapion and his followers, called reasoning from analogy.

From this mode of procedure, it is therefore obvious, that the *experience* of the ancient empiric was founded on the testimony of his senses, corrected by his recollection of what had been observed by others; and on a comparison of what had been ascertained as fact, with what from its not having formerly occurred, must necessarily be the subject of conjecture. So rational were the empirics of ancient days, and so remote from the mischievous ignorance and craft of their modern successors.

Serapion and his adherents exposed the absurdity of attempting to investigate the latent causes of diseases, and strenuously contended that the physician's attention should be wholly confined to such as were obvious to his senses. In the then state of the science, these positions of Serapion and his scholars, were certainly very tenable. It was reserved for anatomy to discover those secret springs of unhealthy action in the system; and it must be remembered, in the time of the Alexandrian Professor, anatomy tomy was in its infancy. They who attempted to explore the latent sources of disorder in the age of Serapion, must have been guided by the glimmering light of the philosophy of the times, a light very inadequate to dissipate the surrounding gloom, or to enable the enquirer to keep aloof from the devious windings of error and of conjecture.

It is obvious, then, that the original founders of empiricism attempted to effect a desirable revolution in medical science. Their object was to rescue the healing art from the dominion of authority, and from groundless hypothesis; a reformation, which discussion relative to the proximate causes of diseases could not accelerate, and they of consequence abandoned this field of inquiry. It were, indeed, morally impossible for them to occupy it with success. They had no certain land-mark to direct them in their researches, they must therefore have groped their way in darkness: they must have substituted chimera for truth, and their erroneous conclusions would instantly lead them to form false indications of cure. To the external or remote causes of diseases the ancient empirics were not inattentive; yet were they not in the least studious to determine the mode of their operation. In no part of their practice were they influenced by the consideration of these causes, and they were objects of attention so far only, as they could be considered as constituting a part of those signs, by which the nature of the disease in question could be successfully ascertained.

The ancient empirics were peculiarly eminent for their talent of observation. With the closest attention they studied the case before then; and in forming a diagnosis, they availed themselves of every fact, with which past experience had stored their memories. Hence the adage, so current among them, that an attentive comparison of the disease under consideration with such similar cases as recollection furnished, constituted the very essence of physic, and comprised all the requisites of successful practice. If they suffered themselves to be influenced by reasoning, in adopting their curative indications, it was by such simple deductions, from the facts under review, as could not possibly lead into error, or by such obvious conclusions, as spontaneously suggested themselves from the circumstances of the case. They therefore proscribed only such reasonings as were founded on false or uncertain principles; they rejected neither the examination of symptoms, nor the phenomena of diseases, nor analogy, nor erudition. The respectable founders of this sect must therefore therefore be exempted from that blame, which so justly attaches to their successors, who propagated tenets which their masters never espoused, and condemned anatomy and physiology, and philosophy, and every branch of collateral science, to which Philenus and Serapion allowed their due weight, and which must, indeed, be regarded as the only permanent basis on which a solid medical superstructure can ever be reared.

From this account of the ancient empirics, which is chiefly extracted from the elegant preface of Celsus to his treatise, "*De re medica*," it will occur to the most superficial observers, how widely they differed both in their principles and in their practice, from their stupid and crafty successors; nor can they indeed be justly charged with the crime of disregarding the dictates of sound reason or of mature reflection.

It is, indeed, well known to such as are conversant with medical history, that to the industry of the ancient empirics we are indebted for the introduction, or rather for the full knowledge of sedative and narcotic remedies, on the liberal exhibitions of which, probably, depended the superior reputation, acquired by some of them over their more cautious antagonists. Of this superiority a singular instance occurs in the many existing testimonies to the fame of Heraclides, of Tarentum, who is handed down to us as the most successful physician whom any age or country ever produced.

But, besides the changes effected by their principles in the essence of medicine, the conduct of the ancient empirics, accomplished a revolution in its external form and condition.

No longer engaged in studying systems, and averse from speculating even on the symptoms of diseases, they exerted their whole faculties, in investigating the power of medical substances, which constituted the basis of their pre-eminence in pharmaceutical skill. When sufficient exercise was found for their peculiar talent as physicians, the dietetical and chirurgical branches of medicine were devolved on other persons, who, in like manner, confined themselves to their several departments.

Thus was it, that the healing art, which hitherto had been uniformly studied and practised, in all its parts, by one man, became divided into various branches, insomuch that the dietetic and pharmaceutic physician seldom acted in concert. The *apotheca* or repository of simple and compound medicines, was, for a long time, in the house of of the pharmaceutic physician, whither they were brought ready for use, by the several tribes of collectors and workmen, who at length availed themselves of such superficial knowledge of their qualities, as they casually collected, to usurp the office and dignity of their masters, in the manner which we shall proceed to describe in a subsequent Number.

*Edinburgh, August* 1, 1805.

[ To be continued. ]

